# Farm-level data on production systems, farmer- and farm characteristics of apple growers in Switzerland

**DOI:** 10.1016/j.dib.2023.109531

**Published:** 2023-08-30

**Authors:** Lucca Zachmann, Chloe McCallum, Robert Finger

**Affiliations:** Agricultural Economics and Policy Group, ETH Zurich, Zurich, Switzerland

**Keywords:** Sustainable agriculture, Pest management, Low-residue, Marketing channels, Pesticide regulation, Information sources, Risk management

## Abstract

We here present survey data from apple growers across Switzerland. Data from 245 apple growers was collected, using an online survey in French and German in 2022. The sampled growers represent 24.4% from total land under apples. Apple production is one of the most economically relevant and pesticide intensive crops. Hence, the focus of the survey is on growers’ pest management decisions and marketing strategies. Survey data contains details on growers’ agronomic practices such as grown cultivars, pest management against fungi, insects, and weeds, as well as pesticide use for cosmetic purposes. Moreover, we collected information on pest management after harvest, i.e. storage loss strategies. Marketing characteristics include the sales channel chosen as well as labels used and contract arrangements with buyers. Moreover, detailed data about farm management strategies, behavioral factors, as well as other farm- and farmer characteristics was collected. Survey data is matched with a rich set of environmental data, i.e. precipitation, temperature, and apple scab infection risk.

Specifications Table

**Table:** 

Subject	Agricultural Economics
Specific subject area	Production systems, Pest management, Marketing channels, Risk preferences, Risk perceptions, Pesticide regulation, Information sources
Type of data	CSV file
How the data were acquired	Online survey using LimeSurvey combined with meteorological data from weather stations.
Data format	Raw Partly filtered (for confidentially reasons)
Description of data collection	The online survey questionnaire was sent out via LimeSurvey to all (N=1’932) apple farmers in German- and French-speaking Switzerland. The questionnaire was available in German and French from July 6, 2022 until August 31, 2022. A total of 245 farmers responded entirely to the survey (response rate: 12.7%). Participation was incentivized. Data was anonymized.
Data source location	- Institution: ETH Zurich - City/Town/Region: Zurich - Country: Switzerland
Data accessibility	Repository name: ETH Zürich Research Collection Data identification number: https://doi.org/10.3929/ethz-b000602779 Direct URL to data: https://doi.org/10.3929/ethz-b-000602779https://www.research-collection.ethz.ch/handle/20.500.11850/602779

## 1 Value of the Data

• Representative country-wide data on detailed farm-level decisions about pest management of apple growers (specifically regarding weeds, insects, fungi, storage diseases and visual appearance of the apples). The data also covers marketing strategies used, farm and farmer characteristics including personality traits such as locus of control and self-efficiency, perceptions about pesticides’ impacts in several areas, and preferences such as time and risk preferences.
• The data enables the examination of pest management decisions at the farm and their many influencing factors (e.g. marketing channels, farmer- and farm characteristics, personality traits, perceptions, and preferences).
• Data also allow the study of farmers’ pesticide regulation perceptions about who should be regulated, who should regulate and how the regulation should be carried out.
• Data allow the analysis of growers' information sources and formats regarding pest management information and the importance of various attributes for source selection.
• Broad range of value to researchers, policy makers, and food-value chain actors to study entry points for sustainable pest management behavior.
• The detailed data collected can be used as standalone database or as comparison with other agricultural crop producers as well as in meta-analyses.

## 2 Objective

Apple production ranks globally among the most economically relevant and pesticide-intensive crops [1,2]. Pest management approaches used differ substantially across farms and farmers. This is of large relevance for food production as well as for the environmental and health impacts of production. Farmers’ choices are for example determined by environmental conditions, policy framework, market conditions as well as farm- and farmer characteristics.

## 3 Data Description

We collected 245 responses from apple growers across Switzerland. The questionnaire was sent to all apple producers in German and French speaking parts of Switzerland, i.e. 1’932 apple growers (response rate: 12.7%).^1^ The questionnaire was available from July 6, 2022, until August 31, 2022.

**Fig. 1:**
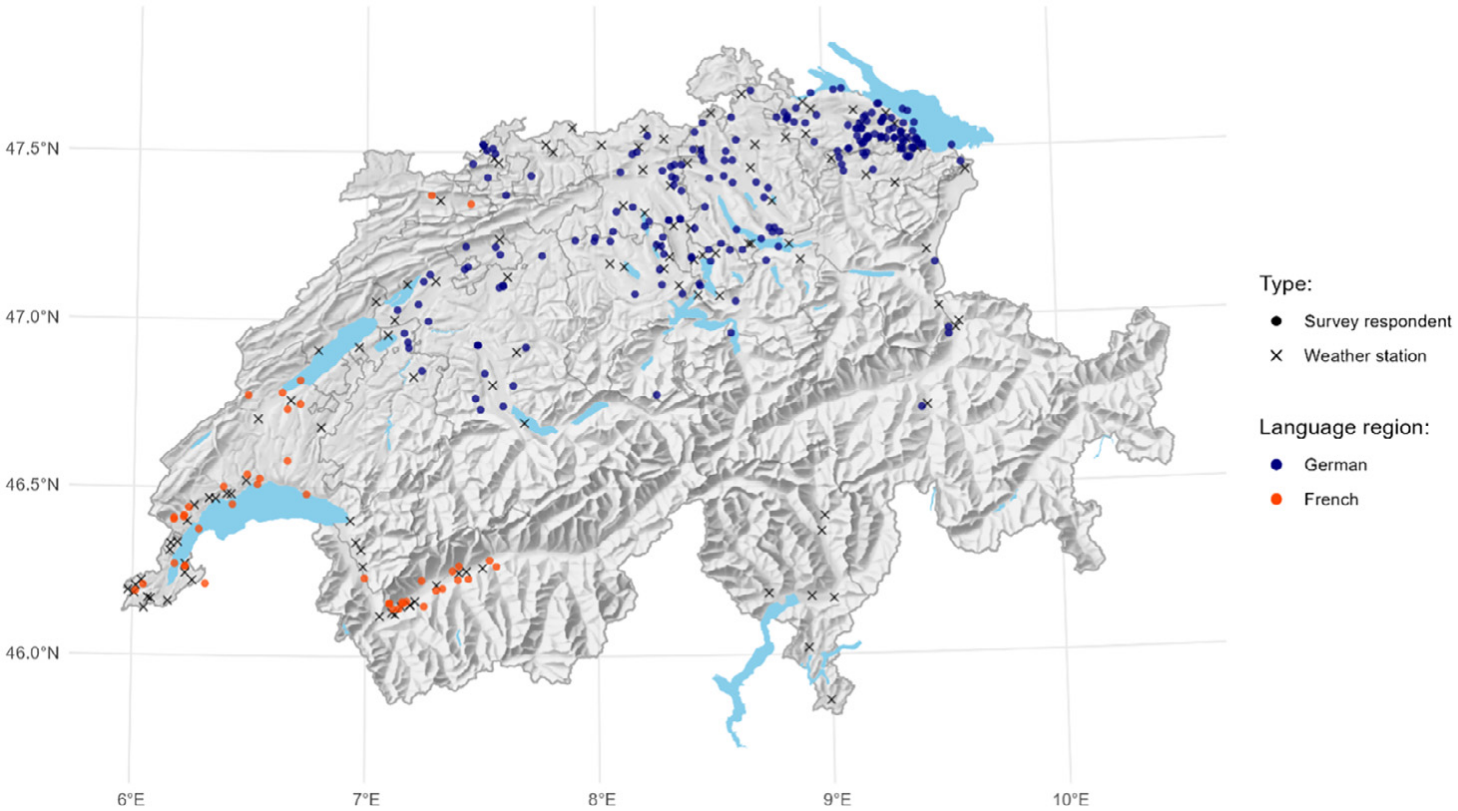
Spatial representation of our sample (N=245). *Note:* The locations of the survey participants are randomly positioned within the municipalities to avoid identification ofdecl farms.

The overarching focus of the data collection was on pest management practices in Swiss apple production. More specifically, the aim of the data collection was to elicit a detailed list of agronomic practices relating to apple production and pest management, as well as a rich set of farmer and farm-level characteristics. We surveyed detailed pest management practices used to control weeds, insects, and fungi. We also focused on strategies against cosmetic damages. Moreover, we survived strategies against storage diseases, i.e. after harvest. The latter two points are expected to be relevant as main reasons for pesticide applications in apple orchards, and pesticide residues as results thereof [4]. Farmer characteristics included socio-demographic variables, perceptions, and farmers’ risk and time preferences. A particular focus of our survey was on marketing strategies. In total, the main survey contained 61 questions. Our survey data were matched with data on environmental factors from weather stations, i.e. temperature, precipitation and regional apple scab infection risk which is the main pest in apple orchards [5]. Fig. 1 presents a spatial visualization of our sample and the weather stations used for nearest-distance matching.

Table 1 shows that our sample is representative for the land covered by apples (24.4% from total land under apples) and specifically land under club varieties. Moreover, the sample is representative in terms of growers’ age and the share of female growers, but slightly overrepresents organic farms. The sample also covers 152 grown varieties (83.4% of all) in Switzerland.

**Table 1:** Sample representativeness.

	Sample	Switzerland	Reference for Switzerland
**Land**			
Land under apples	899 ha (24.4%)	3′686.9 ha	[6]
Land under club varieties	152 ha (19%)	271.5 ha (16.7%)	[7]
			
**Observable characteristics**			
Organic producers	20%	15%	[7]
Female growers	6.9%	6%	[7]
Age	53.1% of farms in our sample were managed by people over 50 years of age	55% of farms in Switzerland were managed by people over 50 years of age	[8]
			
**Varieties**			
Unique identified varieties	152 (83.4%)	181	[7]

*Note:* The values for Switzerland refer to the farming population at large (i.e. different farm types) while our sample focuses specifically on apple producing farms. Refer to Appendix A for the identification of club varieties.

The dataset, survey and codebook describing the variables are available online on the ETH Zürich Research Collection: https://doi.org/10.3929/ethz-b-000602779

## 4 Experimental Design, Materials, and Methods

The survey was carried out via the online platform Limesurvey. We pre-tested the survey with apple growers in the French- and German-speaking part of Switzerland and experts in apple production. To incentivize participation, we offered 50 Swiss Francs to 25 randomly drawn participants. In addition, personalized and aggregate feedback based on the survey results were offered to participants. The median response time was 31 minutes.

The raw data that accompanies this article is structured around the design of the survey which consisted of 6 sections (see Appendix B for the questionnaire):

i) Agronomic practices
ii) Marketing channels and characteristics
iii) Information about the farm
iv) Information about the farm manager
v) Pesticide regulation
vi) Growers’ perceptions, preferences, and personality

### 4.1 Agronomic practices

The survey started with a question about the farms’ size (in are, i.e. 100 square meters). Next, we elicited the cultivars grown. First, we offered participants to choose from a menu of the 16 most grown apple cultivars in Switzerland which varieties the grower cultivates on their farm. Second, in the case that a grower grows different varieties than those listed, we provided space to manually add up to 40 additional varieties. In total, 152 different varieties were indicated, including club or managed varieties.^2^ Moreover, we asked the growers to indicate on what area each variety is grown. Overall, we collected data from 899 ha of land under apples, which is 24.4% from total land under apples in Switzerland [3].

We also asked growers about their agronomic practices regarding apple production and pest management. First, we elicited the production method each farm employs. Answer options were organic, bio-dynamic, or integrated production or according to the ecological performance record. Moreover, growers could indicate other production methods in an open-text field. Second, we asked growers which factors most negatively impact apple yield (i.e. quality and quantity). Hail, fungi, insects, weeds, frost, soil fertility, droughts and excessive rain were proposed answer options, but growers could add individual factors in an open-text field. Third, we zoomed into pest management practices against insects, weeds, and fungi. More specifically, we surveyed a long list of mechanical, preventive, biological, technical, and chemical strategies with which these main pests can be controlled or prevented. Our survey also contained questions on the storage of apples. We elicited whether apples are stored on the farm, and in this case how storage disease is controlled/minimized as this is a relevant issue in apple production [4]. In particular, this contained questions on the methods used against storage diseases such as hot water treatments, deficit irrigation, use of products against storage disease, sorting of infected apples, specific fertilization or through sales management [10,11]. In the case of use of products, we asked for the product names. Fourth, we asked growers about whether they use mechanical methods or chemical products for the main purpose of visual appearance of the apples. More specifically, we asked growers to indicate the method or product they apply. Fifth, we surveyed whether growers’ control/minimize pesticide residues on their apples (i.e. follow a low residue strategy). If they indicated that they do, we asked them how, providing a menu of six strategies (e.g. application of organic products in the second half of the season, use of viruses, resistant varieties or pheromones, use of decision support tools, or considering post-harvest intervals). If they indicated that they do not, we elicited reasons for this. Last, we asked growers what thinning methods they used, ranging from manual (e.g. by hand), to mechanical (e.g. using a machine), to chemical (e.g. growth regulators) or biological (e.g. arnica) thinning methods. We also surveyed the different reasons behind thinning, such as developing good fruit size or color, prevent damage to the tree or regulate alternance [12].

### 4.2 Marketing channels and characteristics

Questions on marketing channels, selling contracts, and labels were also asked. More specifically, we asked growers how they market their apples (i.e. apple types) such as table apples, cider apples, processed apples (e.g. dried or self-pressed), or whether they use the apples for their own consumption. Multiple answers were possible. Thereafter, we asked which share of each apple type is sold to a specific buyer type (i.e. marketing channel). Included buyer types were sales to apple traders, agricultural cooperatives, retail (i.e. regional supermarkets, shops, etc.), direct marketing, other channels (i.e. schools, gastronomy, other farm shops, etc.), or own use. Moreover, for each specific buyer type we elicited whether growers have a contract which is written/formal, oral/informal, or no contract. For table apples, we surveyed which elements are specified in the contract in a binary form (i.e. specified or not), such as delivery quantity, delivery date, visual appearance of the apples, varieties delivered, pesticide residues and price. We also surveyed perceptions of growers relating to marketing strategies following [13] for price uncertainty, ex ante quality requirements, damage caused due to late payment by the buyer, and trust. Lastly, we elicited the labels the farms used to market the apples.^3^

### 4.3 Information about the farm

We surveyed a wide range of farm characteristics, starting with several questions about farm labor. We elicited how many units of standardized workforce the farm employs. Thereafter, we asked growers which type of labor is employed on the farm: family members, permanent employees, short-term or seasonal employees, apprentices, or none. We also surveyed for each employed labor type including the survey responded, what tasks they (i.e. the respondent) carry out on the farm. Considered tasks were orchard work, plant protection, office work, planting decisions, and investment decisions. After questions on labor, we asked growers what share of their land is leased and whether and what kind of insurance they are subscribed to. Thereafter, we also surveyed what federal direct payment programs the farms are enrolled in, and whether they have received subsidies for the purchase of low-drift application machinery. Next, several questions targeted information sources used by the grower. For instance, we asked where they searched for information regarding plant protection (e.g. relating to spray schedule) providing them with ten options plus an open-text field to manually add information sources. For all of the selected sources, we elicited what format the grower used, such as social media, websites, apps, e-mail newsletter, newspapers, printed material, personal contacts, TV/radio, messaging services (e.g. WhatsApp, Telegram) and others. We also asked growers what attributes are important for the selection of information sources such as trust in source, competence of the source, high degree of accuracy of the source, easily applicable information, previous experiences with the source, or similar goals between the source and the grower. Last, we assessed whether the grower is considering any major changes to production practices, pesticide use, or direct payment programs for which more information would be needed.

### 4.4 Information about the farm manager

Information about the growers in the survey included gender, the year of birth, educational background, and status of farm succession. We also asked growers to indicate the share of household income that comes from farming and from apple production, respectively. We asked growers how important specific goals are for their decisions regarding pest management strategies (from very important, important, neither nor, important, very important). The assessed goals were high profitability, high yields, high product quality, high protection for farm workers, low workload, high soil protection, high protection of non-target organisms, and high protection for consumers.

### 4.5 Pesticide regulation

We also elicited various perceptions of growers related to pesticide regulation. First, we asked growers who should regulate pesticides. Answer options included the market (e.g. through retailers, consumers), the state (e.g. through agricultural authorities), farmers (e.g. through the farmers union) and self-regulation (e.g. through branch and producer organizations). Second, we elicited to what extent (from not at all to completely, on a 5-point scale) growers support instruments with which agricultural authorities can regulate pesticides. Assessed instruments were stricter product authorizations and application requirements, direct payments, extension services, promotion of research for pesticide alternatives, labelling of low/no pesticide food products or stakeholder agreements. Last, we asked growers with a multiple-choice question who should be regulated in the context of pesticides. Answer options were pesticide producers, wholesalers, retailers, agricultural extension agents, consumers, or farmers.

### 4.6 Growers’ perceptions, preferences, and personality

We elicited time preferences by asking how willing they are to give up income today in order to benefit more from it in the future [14]. Growers could choose from 0 (not willing) to 10 (very willing). Moreover, we elicited risk preferences using Likert type contextualized self-assessment questions in several domains [15]. The risk preference elicitation was done in 6 different domains: apple production, market and prices, plant protection, agriculture in general, farmers’ health, and environmental protection. Answer options ranged again from 0 (not willing to take risks) to 10 (very willing). In this section, we also elicited several perceptions about low residue production, such as compensation, and feasibility. Additionally, we asked growers how they rate the impact of pesticide use in the areas of product quality, yield, soil protection, water protection, health protection of farmers, and health protection of consumers. Possible answer options ranged from very negative to very positive on a 5-point scale. Last we assessed non-cognitive skills of farmers via Likert type questions [16] by eliciting both locus of control following [17,18] and self-efficacy following [19].

We matched our survey data with spatial environmental data that is of relevance for apple production. We used data from 85 weather station across Switzerland and matched the station data to our observations by minimizing the distance between the two points on the surface of a sphere (i.e. the great-circle-distance). We incorporate simulated apple scab infection risk data according to [20] by averaging station values between 2020 and 2021 which is prior to our data collection. Moreover, we include average temperature and precipitation data at the station level.

## Ethics Statements

We received ethical approval for our study from the Ethics Commission of ETH Zurich on June 1 2022 with approval number 2022-N-89. All participants expressed informed consent prior to entering the survey.

## CRediT authorship contribution statement

**Lucca Zachmann:** Conceptualization, Methodology, Writing – original draft, Visualization. **Chloe McCallum:** Conceptualization, Methodology, Writing – review & editing. **Robert Finger:** Conceptualization, Methodology, Writing – review & editing.

## Data Availability

dataset (Original data) (ETH Research Collection).

## Appendix Supplementary materials

Image, application 1

## Footnotes

1 The French- and German-speaking parts of Switzerland represent 99.93% of the apple-growing land, so we excluded other language regions from our survey [3].

2 Club varieties are patented apples whose production and marketing is planned by the patent right holder [9]. Refer to Appendix A for their definition.

3 Included labels were Bio Suisse (organic), Demeter (bio-dynamic), IP Suisse (integrated production), SwissGAP, Suisse Garantie, Pro Specie Rara (label to promote endangered and rare crops and animals), Club (managed variety), Hochstamm Suisse (label to identify apples from standard trees), or no label.

## References

[1] de Baan L. (2020). https://2020.agrarbericht.ch/de/umwelt/wasser/verkauf-und-einsatz-von-pflanzenschutzmitteln.

[2] Bundesamt für Statistik (BFS) (2021). https://www.bfs.admin.ch/asset/de/18984213.

[3] Böhlen D., Caloz M. (2022). Bundesamt für Landwirtschaft (BLW).

[4] Gölles M., Bravin E., Naef A. (2015). Evaluation of the low-residue apple crop protection. Acta Hortic..

[5] Bowen J.K., Mesarich C.H., Bus V.G.M., Beresford R.M., Plummer K.M., Templeton M.D. (2011). Venturia inaequalis: the causal agent of apple scab: Venturia inaequalis. Mol. Plant Pathol..

[6] Böhlen D., Caloz M. (2023). Bundesamt für Landwirtschaft (BLW).

[7] Widmer C. (2021). https://www.agrarbericht.ch/de/betrieb/strukturen/betriebe.

[8] Bundesamt für Statistik (BFS) (2022). https://www.bfs.admin.ch/bfs/de/home/statistiken/land-forstwirtschaft/landwirtschaft/soziale-aspekte.html.

[9] Legun K.A. (2015). Club apples: a biology of markets built on the social life of variety. Econ. Soc..

[10] Mpelasoka B.S., Behboudian M.H., Dixon J., Neal S.M., Caspari H.W. (2000). Improvement of fruit quality and storage potential of ‘Braeburn’ apple through deficit irrigation. J. Hortic. Sci. Biotechnol..

[11] Maxin P., Weber R., Pedersen H., Williams M. (2012). Hot-water dipping of apples to control Penicillium expansum, Neonectria galligena and Botrytis cinerea: effects of temperature on spore germination and fruit rots. Eur. J. Hortic. Sci..

[12] Dennis F.G. (2000). The history of fruit thinning. Plant Growth Regul..

[13] Hao J., Bijman J., Gardebroek C., Heerink N., Heijman W., Huo X. (2018). Cooperative membership and farmers’ choice of marketing channels – evidence from apple farmers in Shaanxi and Shandong Provinces, China. Food Policy.

[14] Falk A., Becker A., Dohmen T., Enke B., Huffman D., Sunde U. (2018). Global evidence on economic preferences. Quart. J. Econ..

[15] Dohmen T., Falk A., Huffman D., Sunde U., Schupp J., Wagner G.G. (2011). Individual risk attitudes: measurement, determinants, and behavioral consequences. J. Eur. Econ. Assoc..

[16] Wuepper D., Lybbert T.J. (2017). Perceived self-efficacy, poverty, and economic development. Annu. Rev. Resour. Econ..

[17] Rotter J.B. (1966). Generalized expectancies for internal versus external control of reinforcement. Psychol. Monogr..

[18] Abay K.A., Blalock G., Berhane G. (2017). Locus of control and technology adoption in developing country agriculture: evidence from Ethiopia. J. Econ. Behav. Organ..

[19] Bandura A., Pajares F., Urdan T.C. (2006). Self-Efficacy Beliefs of Adolescents.

[20] Werthmüller J., Naef A., Schmitt J., Racca P., Kleinhenz B. (2017). VMVenturia: neues Prognosemodell für den Apfelschorf. Schweizer Zeitschrift Für Obst- Und Weinbau.

