# Supplementary material for: Farm-level data on production systems, farmer- and farm characteristics of apple growers in Switzerland
Source: Data Brief. 2023 Aug 30;50:109531. doi: 10.1016/j.dib.2023.109531 (PMC10502332; doi:10.1016/j.dib.2023.109531)
Supplement: Supplementary file 1 [file mmc1.docx]

**Appendix**

**A Club varieties**

A club variety refers to a specific category of varieties listed and established by the test centre of Laimburg in Italy. The list comprises the most significant club varieties found across Europe, as documented by Guerra [21]. To identify the club varieties cultivated in Switzerland, this list is cross-referenced with the orchard area statistics in Switzerland, as outlined in Böhlen and Caloz [6].

Thus, club varieties are (by brand name): Jazz®, Diwa®, Junami®, Pink Lady®, Greenstar®, Kanzi®, Mairac®, Sweetango®, Tentation®, Kiku®, Rubens®, Envy®, Cameo®, Camela®, Evelina®, Antarès®, Redlove®, Rockit®.

**B Survey questionnaire**

*Ce sondage est également disponible en français (veuillez choisir ci-dessus).Diese Umfrage ist auch in Deutsch verfügbar (bitte oben auswählen).*

Dear apple growers,

We are pleased that you are taking part in this survey financed by the Swiss Science Foundation (SNF) on Swiss apple cultivation. The focus of this survey lies on your current assessment of apple production and marketing in Switzerland. The survey will provide important insights for agricultural practices, extension services and research. Upon request and if you completed the survey in full, we will be happy to generate a report of your individual position in the region and in Switzerland as a whole. In this report you will see how you differ from your neighbours.

The aim of the study is to find out which factors influence the farmers’ decisions regarding management choices, pest management strategies, and distribution channels.

Answering the survey will **take about 25-30 minutes.**

We will also **raffle 25 prizes of Landi vouchers worth 50 CHF**each among all participants who have completed the survey in full.

Thank you very much for your participation!

Best regards,

XXXXXX

If you have any questions, please contact:

XXXXXX
XXXXXX
XXXXXX
XXXXXX

Your participation in the survey is voluntary. Your data and information will of course be treated with strict confidentiality and used exclusively anonymously for scientific purposes. Further information on the conditions of participation and data protection can be found here [link to information sheet for participants].

☐ I hereby confirm that my participation is voluntary and that my anonymized data may be used.

Q0a. Would you like to receive the results of the survey? We’ll send you an individual evaluation.

Yes

No

Q0b. Would you like to participate in the raffle of 25 Landi vouchers valued at CHF 50 each?

Yes

No

# **Section 1: Apple varieties**

Q1. How large is your farm (in are)?*

Q2. Which apple varieties do you cultivate on your farm? * Please only indicate varieties whose cultivated area is at least one are.
Choose the varieties you cultivate. You can add varieties which aren’t listed manually at the end. Please indicate all cultivated varieties.

☐ Gala

☐ Golden Delicious

☐ Braeburn

☐ Scifresh (Jazz®)

☐ Boskoop

☐ Jonagold-Gruppe / Jonagold, groupe

☐ Milwa (Diwa®, Junami®)

☐ Cripps Pink (Pink Lady®)

☐ Rewena

☐ Topaz

☐ Gravensteiner / Gravenstein

☐ Cox Orange

☐ Elstar

☐ Remo

☐ Kanada Reinette / Reinette du Canada

☐ Idared

☐ Add variety manually

☐ Add variety manually

…

Q3. Please indicate the area (in are) devoted to each variety*.

| Varieties | Surface (are) |
| --- | --- |
| [if ticked] |  |
| … |  |

Q4. What percentage of your apple orchard will have been replanted within the next 10 years? _________%

Q5. What are factors you consider relevant when choosing a new variety?

☐ High yield

☐ Disease resistance

☐ Yield stability

☐ Sensory qualities (e.g., acidity, sweetness, taste, bite, etc.)

☐ External qualities (e.g., size, colour, shape, etc.)

☐ Easy care (e.g., low labour input)

☐ High marketing potential

☐ Dealer’s requirements

☐ Picking date (e.g., through harvest staggering)

# **Section 2: Agronomic practices**

Q6. What production method do you use on your farm?

☐ Ökologischer Leistungsnachweis (ÖLN)/Proof of Ecological Performance (PEP)

☐ Organic

☐ Integrated Production (IP-Suisse)

☐ Organic-dynamic

☐ Other: ______

Q7. What factors do you consider to have the biggest negative impact on your orchard yield (quantity and quality)?
Please choose 1 to 2 applicable answers.

☐ Hail

☐ Fungal diseases

☐ Insect related pests

☐ Weeds

☐ Frost

☐ Soil fertility

☐ Drought

☐ Excessive rainfall

☐ Other: _______

Q8. How do you avoid of combat insect pests in your orchard?
Please choose applicable answers. Multiple answers possible:

☐ Confusion techniques (e.g. Pheromones)

☐ Promotion of beneficial insects (e.g. predatory mites, beetles, etc.)

☐ Preventive measures (e.g. Field hygiene, irrigation, plant nutrition, etc.)

☐ Decision support tools (e.g. Early warning systems, prognosis systems, damage threshold systems, etc.)

☐ Use of cultivar mixtures

☐ Insecticides

☐ Mechanical control (e.g. Nets, traps, etc.)

☐ Biological control (e.g. Pyrethrin, spinosad, kaolin, oils, acids, etc.)

☐ Virus compounds (e.g. Granulosis viruses, Bacillus thuringiensis, etc.)

☐ Other: _______

Q9. How do you avoid or control weed in your orchard? Multiple choice
Please choose applicable answers. Multiple answers possible:

☐ Mechanical weeding/Hoeing

☐ Mulching

☐ Herbicides

☐ Other: ________

Q10. How do you avoid or control fungi infestations in your orchard?
Please choose applicable answers. Multiple answers possible:

☐ Remove infected material from the orchard

☐ Decision support tools (e.g. Early warning systems, prognosis systems, damage threshold systems)

☐ Canopy management (e.g. thinning of clusters, air flow control, leaf removal)

☐ Use of cultivar mixtures

☐ Use of rain covers

☐ Use of microorganisms (e.g. *Bacillus subtilis, B. pumilus, Trichoderma* spp., *Fusarium* spp)

☐ Use of inorganic material (e.g. Potassium bicarbonate, Ulmasud, Myco-Sin and Myco-San)

☐ Fungicides

☐ Resistant varieties

☐ Other: _______

Q11. Do you store apples yourself?

☐ Yes

☐ No

Q12. How do you control/prevent damage during storage?

☐ Hot water treatments

☐ Deficit irrigation

☐ Use of products against storage disease

☐ Sorting of apples

☐ Specific fertilization

☐ Sales management (e.g. Choice of sales date)

☐ I do not control/prevent for damage during storage

☐ Other: _______

Q13. Which products do you use for apple storage (e.g. Smartfresh, Blossom Protect, Moon experience, etc.)?

Q14. Do use mechanical methods or plant protection products for the main purpose of visual appearance?
Please name all methods or products you use for this purpose:

☐ Yes, mechanical methods: _____

☐ Yes, plant protection products: ____

☐ No: _____

Q15. Do you control/minimize pesticide residues on the apples?

☐ Yes

☐ No

Q16. If yes, how?

☐ I use only organic pesticides after around mid-June (second half of the season)

☐ Use of living organisms or viruses (e.g. Aurobasidium pullulans, granuloviruses, etc.)

☐ Use of resistant varieties

☐ Pheromones

☐ Use of prognosis models (e.g. RIMPRO) to adopt spay time

☐ Other: _______

Q17. Why not?

☐ Pesticide residues are not relevant for my marketing channels

☐ I do not use pesticides that may cause residues

☐ Other: _______

Q18. What methods for thinning of the apple trees/apple orchards do you apply?

☐ Manual thinning (e.g. by hand, pruning)

☐ Mechanical thinning (e.g. with a spiked-drum shaker)

☐ Chemical thinning (e.g. with plant growth regulators)

☐ I do not thin my apple trees/apple orchards

☐ Other: _______

Q19. Why do you use thinning?

☐ To develop good fruit size

☐ To develop good fruit color

☐ To stabilize apple tree bearing

☐ To prevent damage to the tree (e.g. breaking of limbs, exhaustion of tree reserves)

☐ Other: _______

# **Section 3: Marketing channel**

Q20. How do you market your apples? Multiple options possible.

☐ As dessert apples

☐ As cider apples

☐ As processed apples (e.g. dried, self-pressed, etc.)

☐ I don’t market my apples (Own use)

Q21. How are you marketing your dessert apples (in percent)?

| Sale | Percentage |
| --- | --- |
| Sales to traders |  |
| Sales to cooperatives |  |
| Sales to commerce (e.g. super markets, stores, etc.) |  |
| Direct marketing |  |
| Other channels (e.g. schools, gastronomy, other farm shops, etc.) |  |
| Own use (no sales) |  |

Q22. In what quality class are you marketing your dessert apples (in percent)?

Extra class: ______%

Class I: ______%

Class II: ______%

Q23. How are you marketing your cider apples (in percent)?

| Sale | Percentage |
| --- | --- |
| Sales to traders |  |
| Sales to cideries |  |
| Sales to cooperatives |  |
| Direct marketing |  |
| Own use (no sale) |  |

Q24. How are you marketing your processed apples (in percent)?

| Sale | Percentage |
| --- | --- |
| Sales to traders |  |
| Sales to cooperatives |  |
| Sales to commerce (e.g. super markets, stores, etc.) |  |
| Direct marketing |  |
| Other channels (e.g. schools, gastronomy, other farm shops, etc.) |  |
| Own use (no sale) |  |

Q25. Do you have any type of contract with your buyer(s)?

|  | Written contract / formal | Oral contract / informal | No contract |
| --- | --- | --- | --- |
| Sale of dessert apples to traders |  |  |  |
| Sale of dessert apples to cooperatives |  |  |  |
| Sale of dessert apples to stores |  |  |  |
| Sale of dessert apples to other channels (e.g. schools, gastronomy, other farm stores, etc.) |  |  |  |
| Sale of cider apples to traders |  |  |  |
| Sale of cider apples to cideries |  |  |  |
| Sale of cider apples to cooperatives |  |  |  |
| Sale of processed apples to traders |  |  |  |
| Sale of processed apples to cooperative |  |  |  |
| Sale of processed apples to stores |  |  |  |
| Sale of processed apples to other channels (e.g. schools, gastronomy, other farm shops, etc.) |  |  |  |

Q26. What conditions are specified in your contract for dessert apples?

|  | Yes | No | Don’t know |
| --- | --- | --- | --- |
| Quantity |  |  |  |
| Delivery date |  |  |  |
| Exterior looks of apples |  |  |  |
| Varieties |  |  |  |
| Pesticide residues |  |  |  |
| Price |  |  |  |

Q27. To what extent do you agree with the following statements?

|  | Strongly disagree | Disagree | Neutral | Agree | Strongly agree |
| --- | --- | --- | --- | --- | --- |
| I have no idea about prices offered by buyers beforehand |  |  |  |  |  |
| I know beforehand about the quality requirements of buyers |  |  |  |  |  |
| I suffered loss caused by decisions that were changed unilaterally by buyers during the transaction |  |  |  |  |  |
| I suffered loss caused by delayed payments by buyers |  |  |  |  |  |
| I assume that my buyers have the best intentions. |  |  |  |  |  |

Q28. Do you sell your apples under any of the following labels or designations?
Please choose appropriate answers. Multiple answers possible:

☐ Bio Suisse

☐ Demeter

☐ IP Suisse

☐ SwissGAP

☐ Suisse Garantie

☐ Pro Specie Rara

☐ Club

☐ Hochstamm Suisse

☐ No label

☐ Other: ______

# **Section 4: Information about the farm**

Q29. What’s the focus of your apple orchard? (You can only choose one focus of production.)

Production of club apples

High order quality apples (e.g. Class I)

Second order quality apples (e.g. Class II)

Mixed quality apples

Production for cider apples

☐ Production of apples for juice or dried fruit

☐ Production of apples for own use

Q30. How many units of standard manpower does your farm have? ________

Q31. Do you employ any type of the following labor?

Family members

Permanent workers

Short-term/seasonal workers

Apprentices

None of the above mentioned.

Q32. Which tasks do certain workers do on the farm? **You yourself**

Working in the orchards

Plant protection measures

Office tasks (e.g. marketing, sales, accounting, etc.)

Planting decisions

Investment decisions

Q33. Which tasks do certain workers do on the farm? **Family members**

Working in the orchards

Plant protection measures

Office tasks (e.g. marketing, sales, accounting, etc.)

Planting decisions

Investment decisions

Q34. Which tasks do certain workers do on the farm? **Permanent workers**

Working in the orchards

Plant protection measures

Office tasks (e.g. marketing, sales, accounting, etc.)

Planting decisions

Investment decisions

Q35. Which tasks do certain workers do on the farm? **Short-term/seasonal workers**

Working in the orchards

Plant protection measures

Office tasks (e.g. marketing, sales, accounting, etc.)

Planting decisions

Investment decisions

Q36. Which tasks do certain workers do on the farm? **Apprentices**

Working in the orchards

Plant protection measures

Office tasks (e.g. marketing, sales, accounting, etc.)

Planting decisions

Investment decisions

Q37. Which share of your farmland are you leasing?

○ 0-25% ○ 26-50% ○ 51-75% ○ 76-100%

Q38. Do you have any of the following insurances with Schweizer Hagel Versicherung (Swiss Hail Insurance)?
Please tick all insurances you have.

Basis

Klima

Klima+

I do not have an insurance.

Other: ____

Q39. Which of the following strategies are you employing on your farm?
Multiple answers possible.

Agriculture-related diversification (e.g. agrotourism, dairy farming, crops, etc.)

Working off-farm (e.g. consulting, tourism)

Creation of financial reserves (saving for bad times)

Off-farm investments (e.g. other companies, real estate)

Processing and direct marketing

Pick yourself (i.e. apples are picked by the consumers on the farm)

Forestry work

Other strategies: ______________

Q40. In which direct payment programs does the farm participate?
Please choose correct answers, multiple answers possible:

M1: Partial abandonment of herbicides

M2: Complete abandonment of herbicides

M3: Partial abandonment of fungicides

Standard trees (Hochstamm-Feldobstbäume)

None

Other: ____

Q41. Have you received direct payments for the following purchases/investment of application methods?

☐ 25% acquisition cost for spray blower with horizontal air flow control

☐ 25% acquisition cost for spray blower with horizontal air flow control and vegetation detector

☐ None of the above

Q42. How are plant protection products applied on apple trees/in apple orchards?
Please tick all methods used on the apple trees, multiple answers possible.

Hand sprayer

Tractor without cabin

Closed-cabin tractor

Anti drift nozzle

Spraying equipment with horizontal air assistance

Gun

Tunnel recycling sprayer

Other: ____________________

Q43. Where do you search for plant protection information (e.g. about spraying schedule)?
Select all applicable answers, multiple answers possible.

Cantonal information services (e.g. Kantonale Fachstellen, competence centres, agricultural consultants)

Agroscope

FiBL (Research Institute of Organic Agriculture)

Plant protection product suppliers

Associations (e.g. Farmers association, Swiss fruit association)

Practitioner events

Farmer journals (e.g. Schweizer Bauer, etc.)

Traders

Other farmers/colleagues

I do not search for new information

Other: ___________

Q44. What’s the name of the manufacturer(s) of the plant protection products from whom you get information? __________

Q45. What format of information do you use for plant protection information? Select all applicable.

Social media (e.g. Facebook, Twitter…)

Websites

Apps

E-Mail newsletter

Magazines

Other printed material (e.g. leaflets, flyers)

Personal contact

Television/radio

Short message services (e.g. WhatsApp, Telegram)

Other: ___________

Q46. What is important for you when choosing an information source? Select all applicable.

Trust in source

Expertise of source

High level of accuracy

Information can be readily applied.

Previous experience with the information source

Information source has similar objectives to me

Other: ___________

Q47. Are you currently considering any major changes to the mode of production, use of plant protection products or direct payment schemes for which you would need further information? Select all applicable.

Yes, I am considering changing production mode

Yes, I am considering changing usage of plant protection products

Yes, I am considering changing direct payment schemes

No

# **Section 5: Information about the participant**

Q48. Your sex

Female

Male

Q49. In which year were you born (JJJJ)? ____________

Q50. Do you have formal training in agriculture from any of the following? Multiple choice

EFZ (e.g. Obstfachmann/frau, etc.)

Agricultural management school/ "Meister" degree

Higher technical school (HF)

University of applied science

University or ETH

Fachbewilligungskurs Pflanzenschutz

Degree of Landwirt

Further education in plant protection

Other: ___________

Q51. Do you have a successor to your farm?

Yes

Rather yes

Rather no

No

No, there’s no farm succession in the next 15 years

No, the farm will be discontinued

Q52. Which percentage of your earnings are from farming?

○ 0% ○ 1-25% ○ 26-50 % ○ 51 - 75 % ○ 76 – 100%

Q53. How much is the apple production contributing to your farming income (in percent)?

○ 0% ○ 1-25% ○ 26-50 % ○ 51 - 75 % ○ 76 – 100%

Q54. How important is each objective for your pest management decisions?

|  | Totally irrelevant | Mostly irrelevant | Neutral | Mostly important | Very important |
| --- | --- | --- | --- | --- | --- |
| High profitability | ☐ | ☐ | ☐ | ☐ | ☐ |
| High yields | ☐ | ☐ | ☐ | ☐ | ☐ |
| High quality produce | ☐ | ☐ | ☐ | ☐ | ☐ |
| High health protection of field workers | ☐ | ☐ | ☐ | ☐ | ☐ |
| Low workload | ☐ | ☐ | ☐ | ☐ | ☐ |
| High soil protection | ☐ | ☐ | ☐ | ☐ | ☐ |
| High protection towards non-target organisms | ☐ | ☐ | ☐ | ☐ | ☐ |
| High consumer health protection | ☐ | ☐ | ☐ | ☐ | ☐ |

# **Section 7: Regulation**

Q55. Who do you think should be in charge of pesticide regulation?

The market (e.g. retailers, consumers)

Government (e.g. Federal Office of Agriculture,…..)

Farmers (e.g. Farmers’ association)

Self regulation (e.g. industry organisations, producer organisations)

Q56. The federal government and the cantons have various measures at their disposal to regulate the use of plant protection products. Please indicate your support for the following measures.

|  | Not at all | Mostly not | Neither agree nor disagree | Mostly agree | Completely agree |
| --- | --- | --- | --- | --- | --- |
| Stricter approval and application regulations |  |  |  |  |  |
| Direct payments to promote PPP-reduced / free production |  |  |  |  |  |
| Expansion of the range of consulting / training services and the obligation to provide further training for users |  |  |  |  |  |
| Promote research on alternative PPPs / production systems and protective measures. |  |  |  |  |  |
| Labels for PPP-reduced / free foods for identification and traceability of production and quality criteria |  |  |  |  |  |
| Stakeholder gentlemen’s agreements (e.g. an informal and legally non-binding agreement) |  |  |  |  |  |

Q57. Who do you think should be regulated regarding pesticides?

Pesticide producers

Traders

Retailers

Farm advisors

Consumers

Farmers

Others: _______

# **Section 8: Perceptions and preferences**

Q58. How willing are you to give up income that is beneficial for you/the farm today in order to benefit more from that in the future?

| not willing |  |  |  |  |  |  |  |  |  | very willing |
| --- | --- | --- | --- | --- | --- | --- | --- | --- | --- | --- |
| 0 | 1 | 2 | 3 | 4 | 5 | 6 | 7 | 8 | 9 | 10 |
|  |  |  |  |  |  |  |  |  |  |  |

Q59. Are you willing to take risks or do you try to mitigate risks in the areas mentioned below?

*Please indicate on the scale below. The value of 0 stands for “not willing to take a risk at all” and the value 10 for “very willing to take a risk”. With the values in between you can grade your assessment.*

| Apple production | \| not willing \|  \|  \|  \|  \|  \|  \|  \|  \|  \| very willing \| \| --- \| --- \| --- \| --- \| --- \| --- \| --- \| --- \| --- \| --- \| --- \| \| 0 \| 1 \| 2 \| 3 \| 4 \| 5 \| 6 \| 7 \| 8 \| 9 \| 10 \| \|  \|  \|  \|  \|  \|  \|  \|  \|  \|  \|  \| \|  \|  \|  \|  \|  \|  \|  \|  \|  \|  \|  \| |
| --- | --- | --- | --- | --- | --- | --- | --- | --- | --- | --- | --- | --- | --- | --- | --- | --- | --- | --- | --- | --- | --- | --- | --- | --- | --- | --- | --- | --- | --- | --- | --- | --- | --- | --- | --- | --- | --- | --- | --- | --- | --- | --- | --- | --- | --- |
| Market and prices | \| not willing \|  \|  \|  \|  \|  \|  \|  \|  \|  \| very willing \| \| --- \| --- \| --- \| --- \| --- \| --- \| --- \| --- \| --- \| --- \| --- \| \| 0 \| 1 \| 2 \| 3 \| 4 \| 5 \| 6 \| 7 \| 8 \| 9 \| 10 \| \|  \|  \|  \|  \|  \|  \|  \|  \|  \|  \|  \| \|  \|  \|  \|  \|  \|  \|  \|  \|  \|  \|  \| |
| Plant protection | \| not willing \|  \|  \|  \|  \|  \|  \|  \|  \|  \| very willing \| \| --- \| --- \| --- \| --- \| --- \| --- \| --- \| --- \| --- \| --- \| --- \| \| 0 \| 1 \| 2 \| 3 \| 4 \| 5 \| 6 \| 7 \| 8 \| 9 \| 10 \| \|  \|  \|  \|  \|  \|  \|  \|  \|  \|  \|  \| \|  \|  \|  \|  \|  \|  \|  \|  \|  \|  \|  \| |
| Agriculture in general | \| very willing \|  \|  \|  \|  \|  \|  \|  \|  \|  \| very willing \| \| --- \| --- \| --- \| --- \| --- \| --- \| --- \| --- \| --- \| --- \| --- \| \| 0 \| 1 \| 2 \| 3 \| 4 \| 5 \| 6 \| 7 \| 8 \| 9 \| 10 \| \|  \|  \|  \|  \|  \|  \|  \|  \|  \|  \|  \| \|  \|  \|  \|  \|  \|  \|  \|  \|  \|  \|  \| |
| Your health | \| very willing \|  \|  \|  \|  \|  \|  \|  \|  \|  \| very willing \| \| --- \| --- \| --- \| --- \| --- \| --- \| --- \| --- \| --- \| --- \| --- \| \| 0 \| 1 \| 2 \| 3 \| 4 \| 5 \| 6 \| 7 \| 8 \| 9 \| 10 \| \|  \|  \|  \|  \|  \|  \|  \|  \|  \|  \|  \| \|  \|  \|  \|  \|  \|  \|  \|  \|  \|  \|  \| |
| Environmental protection | \| very willing \|  \|  \|  \|  \|  \|  \|  \|  \|  \| very willing \| \| --- \| --- \| --- \| --- \| --- \| --- \| --- \| --- \| --- \| --- \| --- \| \| 0 \| 1 \| 2 \| 3 \| 4 \| 5 \| 6 \| 7 \| 8 \| 9 \| 10 \| \|  \|  \|  \|  \|  \|  \|  \|  \|  \|  \|  \| \|  \|  \|  \|  \|  \|  \|  \|  \|  \|  \|  \| |

Q60. How do you rate the effect of plant protection products use on the following areas?

|  | Very negative | Negative | Neither negative or positive | Positive | Very positive |
| --- | --- | --- | --- | --- | --- |
| Quality of production | ☐ | ☐ | ☐ | ☐ | ☐ |
| Soil protection | ☐ | ☐ | ☐ | ☐ | ☐ |
| Yield | ☐ | ☐ | ☐ | ☐ | ☐ |
| Water protection | ☐ | ☐ | ☐ | ☐ | ☐ |
| Farmers’ health protection | ☐ | ☐ | ☐ | ☐ | ☐ |
| Consumers’ health protection | ☐ | ☐ | ☐ | ☐ | ☐ |

Q61. Do you agree or disagree with the following statements about your attitudes towards apple production?

|  | Strongly disagree | Disagree | Neither agree or disagree | Agree | Strongly agree |
| --- | --- | --- | --- | --- | --- |
| When I encounter difficulties in apple production, I can usually think of a solution | ☐ | ☐ | ☐ | ☐ | ☐ |
| I am confident that I can accomplish my production goals at the end of the harvest | ☐ | ☐ | ☐ | ☐ | ☐ |
| I can solve production issues if I invest the necessary effort | ☐ | ☐ | ☐ | ☐ | ☐ |
| How successful my apple production is depends mostly on my skills as a farmer | ☐ | ☐ | ☐ | ☐ | ☐ |
| Apple growing is more dependent on the weather than on what I do | ☐ | ☐ | ☐ | ☐ | ☐ |
| Success in apple production can only be slightly influenced by farmers | ☐ | ☐ | ☐ | ☐ | ☐ |
| I usually set myself quite ambitious production goals | ☐ | ☐ | ☐ | ☐ | ☐ |

# **Concluding question**

Q62. Has the last year of production been different to the last five years before? If yes, in what regard?

Q63. Do you have any comments/feedback in general? __________________________

Q64. You have agreed to receive the results of the survey and/or take part in the raffle for the 50 CHF Landi voucher. Please write your e-mail address and/or mailing address in order for us to send you the results and/or the voucher in case you win.

e-mail address: _____________

First name: ___________

Last name: __________

Street: ____________

Street number: ___________

Postcode: _________

City: ________

Thank you very much for your participation. We will treat your personal data as strictly confidential. If you have any questions or comments, please contact:

XXXXXX
XXXXXX
XXXXXX

If you’ve left us your e-mail address and asked for an individual feedback, we will send you the feedback. If you are the lucky winner of the draw, we will notify you of course.

You can now close the Internet browser.
